# Preclinical Evaluation of ^89^Zr-Df-IAB22M2C PET as an Imaging Biomarker for the Development of the GUCY2C-CD3 Bispecific PF-07062119 as a T Cell Engaging Therapy

**DOI:** 10.1007/s11307-021-01621-0

**Published:** 2021-06-18

**Authors:** Kevin P. Maresca, Jianqing Chen, Divya Mathur, Anand Giddabasappa, Adam Root, Jatin Narula, Lindsay King, David Schaer, Jonathan Golas, Keith Kobylarz, Edward Rosfjord, Edmund Keliher, Laigao Chen, Sripad Ram, Eve H. Pickering, James S. Hardwick, Paul A. Rejto, Amira Hussein, Ohad Ilovich, Kevin Staton, Ian Wilson, Timothy J. McCarthy

**Affiliations:** 1 Worldwide Research, Development & Medicine, Pfizer Inc, New York, USA; 2 Regneron Pharmaceuticals, Tarrytown, NY USA; 3 Generate Biomedicines, Inc, Cambridge, MA USA; 4 Black Diamond Therapeutics, New York, NY USA; 5 Invicro, A Konica Minolta Company, New Haven, USA; 6 Evergreen Theragnostics, Jersey City, NJ USA; 7 Memorial Sloan Kettering Cancer Center, New York, NY USA; 8 ImaginAb Inc., Inglewood, CA USA

**Keywords:** ^89^Zr-IAB22M2C PET imaging, CD8 T cell, GUCY2C bispecific antibody, Immuno-oncology

## Abstract

**Purpose:**

A sensitive and specific imaging biomarker to monitor immune activation and quantify pharmacodynamic responses would be useful for development of immunomodulating anti-cancer agents. PF-07062119 is a T cell engaging bispecific antibody that binds to CD3 and guanylyl cyclase C, a protein that is over-expressed by colorectal cancers. Here, we used ^89^Zr-Df-IAB22M2C (^89^Zr-Df-Crefmirlimab), a human CD8-specific minibody to monitor CD8+ T cell infiltration into tumors by positron emission tomography. We investigated the ability of ^89^Zr-Df-IAB22M2C to track anti-tumor activity induced by PF-07062119 in a human CRC adoptive transfer mouse model (with injected activated/expanded human T cells), as well as the correlation of tumor radiotracer uptake with CD8+ immunohistochemical staining.

**Procedures:**

NOD SCID gamma mice bearing human CRC LS1034 tumors were treated with four different doses of PF-07062119, or a non-targeted CD3 BsAb control, and imaged with ^89^Zr-Df-IAB22M2C PET at days 4 and 9. Following PET/CT imaging, mice were euthanized and dissected for *ex vivo* distribution analysis of ^89^Zr-Df-IAB22M2C in tissues on days 4 and 9, with additional data collected on day 6 (supplementary). Data were analyzed and reported as standard uptake value and %ID/g for *in vivo* imaging and *ex vivo* tissue distribution. In addition, tumor tissues were evaluated by immunohistochemistry for CD8+ T cells.

**Results:**

The results demonstrated substantial mean uptake of ^89^Zr-Df-IAB22M2C (%ID/g) in PF-07062119-treated tumors, with significant increases in comparison to non-targeted BsAb-treated controls, as well as PF-07062119 dose-dependent responses over time of treatment. A moderate correlation was observed between tumor tissue radioactivity uptake and CD8+ cell density, demonstrating the value of the imaging agent for non-invasive assessment of intra-tumoral CD8+ T cells and the mechanism of action for PF-07062119.

**Conclusion:**

Immune-imaging technologies for quantitative cellular measures would be a valuable biomarker in immunotherapeutic clinical development. We demonstrated a qualification of ^89^Zr-IAB22M2C PET to evaluate PD responses (mice) to a novel immunotherapeutic.

**Supplementary Information:**

The online version contains supplementary material available at 10.1007/s11307-021-01621-0.

## Introduction

In the expanding field of immuno-oncology (IO), there is a growing need for earlier and more accurate *in vivo* molecular markers that can measure immune responses to an increasing number of IO therapies (IOT). A tool of this nature would also have potential to guide clinical management of disease for approved interventions [1]. Current standard-of-care evaluations rely on either biopsy or imaging-based morphological measurements [2] with the latter approach requiring an extended timeframe (8–12 weeks) to manifest. In addition, the phenomenon of pseudo-progression (a response after progression) may confuse radiologists, delaying formal treatment decisions and conclusions regarding disease evaluation [3].

Positron emission tomography (PET) is a well-established non-invasive imaging technique that has the sensitivity to detect changes in biological processes at the molecular level and has expanded to include ImmunoPET, which employs antibody-based radiotracers to image tumors based on expression of tumor-associated antigens [4–6]. PET tracers contain positron-emitting radionuclides that can be incorporated into a variety of molecular targeting compounds (*e.g.*, small molecules, peptides, antibodies, nanoparticles). PET imaging is a quantifiable and clinically translatable technique, most widely used in clinical oncology for detection of tumors and staging of disease [7]. PET tracers can be delivered at sub-pharmacological doses, and are biologically indistinguishable from their stable natural counterpart, allowing them to image with limited safety concerns and minimal disturbance of the biological system being monitored. PET imaging can impact treatment decisions in IO through the ability to perform whole-body imaging, potentially directing biopsy, and identifying lesions that are either responding or not responding to therapy.

Gastrointestinal malignancies, including colorectal cancer (CRC), gastric cancer, and esophageal cancer, continue to be areas of high unmet medical need despite advances in targeted therapies [8]. CRC affects both men and women and will be responsible in 2020 for an estimated 1,800,977 cancer diagnoses in adults worldwide, and 9.1% of all cancer deaths [9]. PF-07062119 is a T cell engaging BsAb targeting guanylyl cyclase C (GUCY2C), a protein that is over-expressed in colorectal cancers (CRC) and other gastrointestinal malignancies (8). GUCY2C expression in normal tissues is restricted to the apical side of the intestinal epithelium and is broadly expressed in > 90% of colorectal adenocarcinomas across all stages [10, 11]. This BsAb, PF-07062119, is designed to directly synapse T cells with GUCY2C-expressing cancer cells, by simultaneously binding one arm to the tumor-associated cell surface antigen and the other arm to the CD3ε protein on T cells. The formation of productive BsAb-dependent synapses between tumor cells and CD8+ T cells, which play a critical role in immune defense against intracellular pathogens as well as in tumor surveillance [12, 13], can lead to cytotoxic responses directed against the tumor. Previous studies have demonstrated the efficacy of PF-07062119 as a targeted immuno-therapeutic candidate in human CRC xenograft mouse models with adoptive transfer of human T cells [14]. Specifically, PF-07062119 showed potent T cell–mediated *in vitro* activity and *in vivo* efficacy in multiple human colorectal cancer xenograft tumor models, demonstrating that GUCY2C-positive tumors can be targeted with an anti-GUCY2C/anti-CD3 BsAb, with selective drug biodistribution to tumors. Currently, PF07062119 is in the clinic as a potential BsAb treatment of gastrointestinal malignancies (NCT04171141).

The goal of this research was to demonstrate that by imaging CD8+ tumor infiltrating T cells using radiolabeled antibody fragments it would be possible to evaluate early responses to immunotherapies [15]. ^89^Zr-Df-IAB22M2C is a humanized CD8 specific minibody developed by ImaginAb Inc. (Inglewood CA) conjugated with desferrioxamine (Df) and radiolabeled with [^89^Zr] [16]. The minibody (Mb) maintains the specificity of full-length antibodies but has no immune effector functions and is biologically inert. ^89^Zr-Df-IAB22M2C completed phase 1 clinical testing (NCT03107663) in patients with solid malignancies or Hodgkin’s lymphoma [17] and is currently under testing in a phase 2 clinical study (NCT03802123) in patients with metastatic solid tumors.

In this report, we investigated the ability of ^89^Zr-Df-IAB22M2C to track time- and dose-dependent anti-tumor responses induced by PF-07062119 in comparison to treatment with a non-targeted control CD3 BsAb. Experiments utilized a human CRC adoptive transfer mouse model employing *in vivo* PET imaging and *ex vivo* gamma counting, as well as correlation of the radiotracer uptake to CD8 immunohistochemical (IHC) staining and flow cytometry.

## Materials and Methods

### Generation of Anti-GUCY2C Antibodies

Anti-GUCY2C antibodies were generated using hybridoma technology as previously described [14] leading to PF-07062119.

### PBMC Collection and Isolation and Expansion of Human T Cells

Whole blood was collected from healthy donors and immediately treated with the anti-coagulant 10 mM ethylenediaminetetraacetic acid (EDTA). All peripheral blood mononuclear cell (PBMC) and T cell isolations were carried out at room temperature. Each 25 ml of blood was mixed with 10 ml of Dulbecco’s phosphate-buffered saline (DPBS) containing 2 mM EDTA and layered above the frit of a 50-ml Accuspin tube pre-loaded with 15 ml of Histopaque-1077 density gradient media (Sigma-Aldrich, Saint Louis, MO). Tubes were spun at 800×*g* for 20 min, and the material above the frit was decanted into a fresh 50-ml conical tube and spun again at 800×*g* for 10 min. Following centrifugation, the supernatant was discarded, and the pellet was resuspended in DPBS containing 2 mM EDTA. PBMCs were spun at 200×*g* for 10 min, the supernatant was discarded, and the cells were resuspended to 5×10^7^ cells/ml in Robosep buffer. T cells were isolated using the EasySep human T cell enrichment kit (Stem Cell Technologies, Arlington, VA) according to the manufacturer’s protocol. T cells were activated using a Human T cell activation/ Expansion kit (Miltenyi Biotech, San Diego, CA) following the manufacturer’s protocol using T cell media composed of X-Vivo 15 with 5% human serum AB, 1% Penn/Strep, and 0.01 mM 2-mercaptoethanol (Sigma-Aldrich, Saint Louis, MO). After 48 h of T cell activation, T cells were transferred to a G-Rex cell culture device (Wilson Wolf Manufacturing Corporation, New Brighton, MN) for expansion, and human IL-2 (Shenandoah Biotechnology Inc., Warwick, PA) was added to the media at a final concentration of 5 ng/ml and replenished after 2 days. T cells were harvested after 5 days of expansion. At the time of harvest, beads were removed with a magnet, and cells were resuspended in DPBS at 1×10^7^ cells/ml for *in vivo* studies.

### LS1034 Xenograft Tumor Model and BsAb Treatment

Female NOD-SCID IL-2Rγ null (NSG) mice (Jackson Laboratory Bar Harbor, ME) were used for experiments (n = 6 / group) under a protocol approved by an Institutional Animal Care and Use Committee. For xenograft studies, individual NSG mice were implanted in the flank with 5 × 10^6^ LS1034 cells in a total injection volume of 0.2 ml that contained 50% Matrigel Basement Membrane Matrix (Trevigen, Gaithersburg, MD).

Approximately 2 weeks after implantation, when average tumor size per group reached approximately 200 mm^3^, activated/expanded human T cells (2 ×10^6^ cells in 100 μl) were injected intravenously into each mouse (designated as day −1, one day prior to BsAb treatment initiation defined as day 0). On day 0 (24 h after injecting T cells), PF-07062119 or a non-targeted CD3 control BsAb (PF-07079699) was administered intravenously to each animal in a volume of 200 μl. PF-07062119 was formulated at different concentrations to achieve dose levels of 0.03 mg/kg, 0.06 mg/kg, 0.1 mg/kg, and 1.0 mg/kg, while the PF-07079699 was formulated to achieve a dose of 1.0 mg/kg. BsAb treatment was administered intravenously again on day 7 to groups of mice for which the imaging analysis was conducted at day 9 following treatment start.

### PET/CT Imaging and Tissue Assessments

The radiotracer used in the study (^89^Zr-Df-IAB22M2C) was prepared and provided by Memorial Sloan Kettering Cancer Center (New York, NY). HPLC analysis of ^89^Zr-Df-IAB22M2C on injection days demonstrated doses provided were at a radiochemical purity (RCP) greater than 94%, with a specific activity of 2 mCi/mg. On day 3 or day 8, 48 – 81 μCi ^89^Zr-Df-IAB22M2C was administered to each animal intravenously to deliver approximately 50 μg in a volume of 190 – 210 μl. A 30-min static PET imaging in 4-bed hotel was conducted at 22 h post-tracer injection followed by a CT imaging for anatomical registration. The optimal imaging window at 22 h was determined by considering both half-life and pharmacokinetics of the tracer. After imaging, animals (n = 6 / group) were euthanized, the tissues of interest collected, and weighed and their radioactivity counted using a gamma counter (Wallac Wizard 1470). The percentage of the injected dose of the radioactivity per gram tissue (%ID/g) and/or percentage of the injected dose of the radioactivity per tissue/organ (%ID) were calculated. The tissues of interest in this study included whole blood, plasma, tumor (half), brain, lungs (both), liver, spleen, kidneys (both), muscle, small intestine (emptied), large intestine (emptied), skin, muscle, and tail (site of injection). After imaging, the tissues were collected in pre-weighed gamma counting tubes containing the required amount of 10% neutral buffered formalin for gamma counting and later IHC. Additionally, tumor tissues from selected groups were subject to cell extraction for flow cytometry analysis (as described in the supplementary methods).

### Imaging Analysis

Reconstructed images from the Siemens™ Inveon PET/CT were generated in units of activity. Namely, the values assigned to the voxels (volume elements) comprising the 3D reconstructed PET images were in units of μCi. Reconstructed PET and CT images were co-registered, resampled to 0.2-mm^3^ isotropic voxels, and then split using bounded cylinders to separate out each animal’s respective PET and CT data. The PET and CT image registration was confirmed, and images were cropped to a uniform size using VivoQuant (version 4.0) software prior to analysis. Regions of interest (ROIs) were created for the left ventricle, brain, heart, lungs (both), liver, spleen, kidneys, and muscle (quadriceps) by fitting ellipsoids of fixed volume to the respective organs in each image. The tumor and tail ROIs were hand-drawn utilizing the CT image and PET signal. Group and individual master spreadsheets were generated which included the activity (μCi) at the 22-h time point for each ROI generated as well as %ID/g. All PET images were converted to units of %ID/g and shown with the color bar range of 0–25%. CT images were co-registered and shown overlaid with the PET image. Maximum intensity projections (MIPs) were generated for all subjects, as well as montaged images based on group. All images were generated using VivoQuant (Invicro, Boston, MA).

### CD8 Immunohistochemistry

All immunohistochemistry assays were performed on FFPE sections for CD8, similarly to those previously described (14). For image acquisition, slides were scanned on a Leica/Aperio AT2 whole-slide digital scanner using the ×20 magnification setting. Images were saved in .svs format. Whole-slide image analysis of CD8 IHC images was carried out using Visiopharm 7.02 software. The viable tumor regions were manually annotated, and the viable tumor area was calculated using the calculate Area APP. A custom APP was developed to detect and count CD8+ cells. All statistical analyses were carried out in GraphPad Prism 7.02 software. A non-parametric test (Kruskal-Wallis test with Dunn’s multiple comparison) was used to assess for statistical significance at 95% level of confidence among the different treatment groups.

### Statistical Analysis (PET Imaging and Gamma Counting)

Statistical tests were performed using Python (Python Software Foundation, Wilmington, DE). Pairwise comparisons were performed to assess differences between time points within each treatment group and among treatment groups for each time point. False discovery rate correction was applied to the resulting *p*-values. Adjusted *p*-values less than 0.05 were reported as significant. To avoid a loss of statistical power due to multiple comparison’s correction, groups were only compared to groups at the same time point, or groups with the same dose level at a different time point.

## Results

### Robust Adoptive Transfer Model Employed for 89Zr-Df-IAB22M2C PET Qualification Studies

In this study, we evaluated the uptake of ^89^Zr-Df-IAB22M2C in a CRC LS1034 tumor-bearing mice inoculated with activated T cells, which were subsequently treated with either GUCY2C-CD3 bispecific antibody PF-07062119 (a novel T cell engaging bispecific antibody treatment for GUCY2C expressed tumors (Figure S1)) or a non-targeted-CD3 bispecific control PF-07069699 (one or two doses) as previously studied [14, 18], using PET/CT imaging and gamma counting. A schematic of the executed study plan is shown in Figure S2. Significant dose- and time-related differences were observed in comparison to control groups. Results were analyzed as comparisons between imaging days (day 4 versus day 9), as well as between treatment groups.

### *In Vivo* CD8 PET Imaging Reveals Dose- and Time-Dependent T Cell Recruitment

**Fig. 1.**
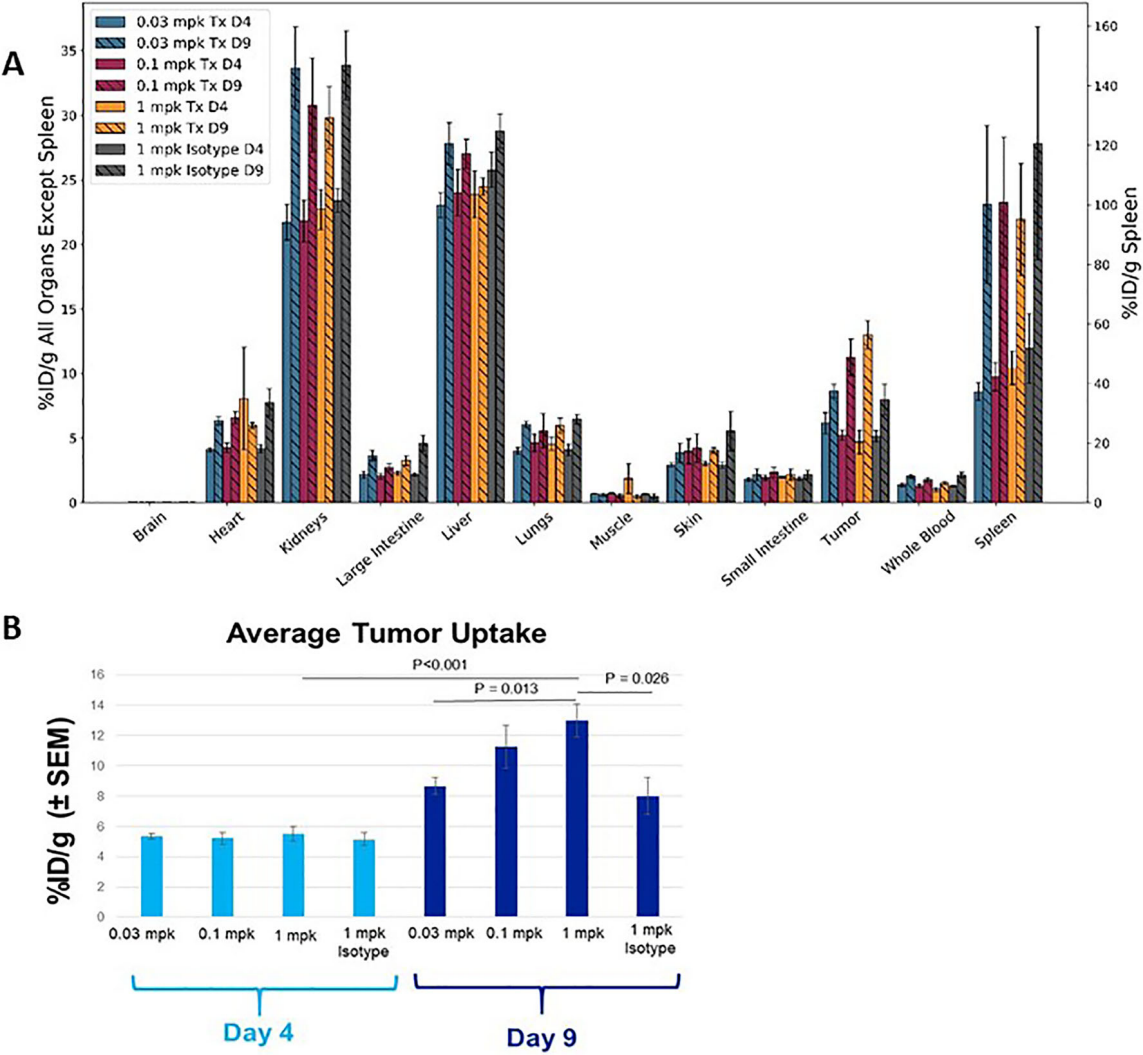
(**a**) *Ex vivo* gamma counting of selected tissue biodistribution and (**b**) tumors of LS1034 mice (mean %ID/g ± SEM) at 22 h post ^89^Zr-Df-IAB22M2C injection on days 4 and 9 following initiation of BsAb treatment 0.03, 0.1, and 1 mpk for PF-07062119 (Tx) and 1 mpk for PF-07079699 non-targeted CD3 (Isotype) control treated.

**Fig. 2.**
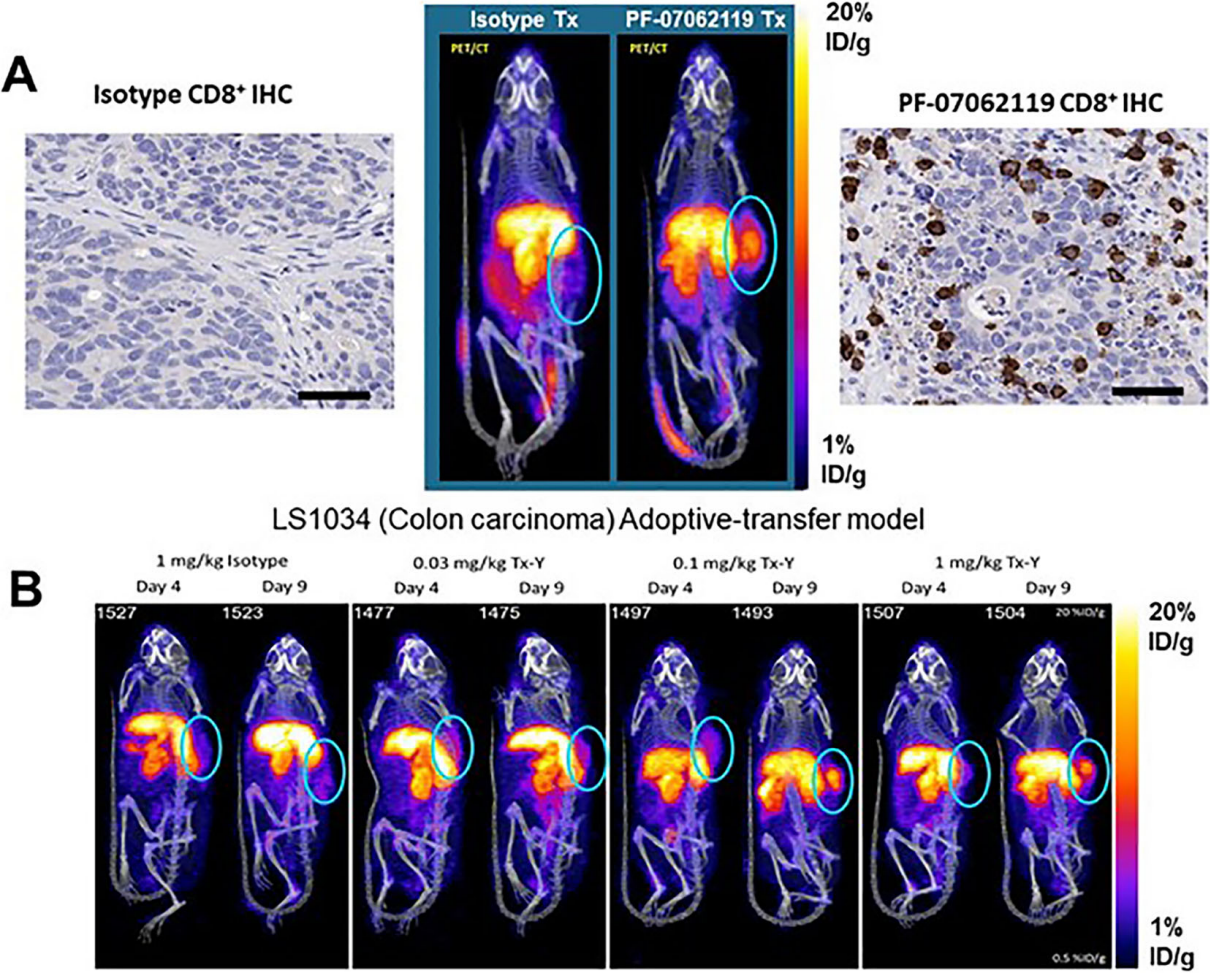
(**a**) Representative PET/CT images at 22 h post-injection of ^89^Zr-Df-IAB22M2C on day 9: 1 mg/kg isotype treated (PF-07069699, left) and GUCY2C BsAb treated (PF-07062119, right), with respective CD8+ T staining (brown; IHC, scale bar equal 50 μm). (**b**) Representative PET/CT images at 22 h post-injection of ^89^Zr-Df-IAB22M2C exploring differences in temporal and dose response effects.

The whole-body distribution of the Immuno-PET tracer demonstrated uptake in CD8 T cell–rich tissues of the spleen and tumor, as well as the primary clearance organs (the liver and kidneys) are shown in Fig. 1A and B. Minimal accumulation of radiotracer was observed in background tissues, such as muscle and whole blood. Specifically, ^89^Zr-Df-IAB22M2C demonstrated significant radioactive uptake on day 9 in CD8 T cell–rich tissues 22 h after injection; this was most evident in the spleen and xenograft tumors on the flank of the animals as depicted in Fig. 2. The accumulation of the radioactivity in the tumors of the PF-07062119–treated animals all increased as a function of dose and time, with a mean SUV of up to 1.54 ± 0.10 (SEM) obtained at 1 mg/kg PF-07062119 dosing at day 9. The animals treated with isotype control had a slight visual tumor uptake, but this accumulation was diffuse, and remained constant over the time period evaluated, similar to background tissues such as muscle (Fig. 2B). Evaluating the day 4 results, there were no significant differences in the measured *in vivo* tumor concentrations between the dose groups. The tumor uptake was unremarkable, suggesting that the day 4 timepoint may have been too early to observe any significant immune response. In contrast, *in vivo* PET imaging data indicated dose-dependent differences between the dose groups on day 9. Specifically, for PF-07062119, there were significant differences between the two higher dose levels (0.1 and 1 mg/kg), and the lowest dose level (0.03 mg/kg), with *p* = <0.001 and p = 0.006, respectively. Tumor imaging by PET also revealed a significantly higher ^89^Zr-Df-IAB22M2C signal on day 9 relative to day 4 for the 0.1 mg/kg and 1 mg/kg PF-07062119-dosed animals (*p* < 0.001 and *p* = 0.001, respectively), as shown in Table 1.

**Table 1.** Tumor uptake of ^89^Zr-Df-IAB22M2C in all groups as percent injected dose per gram (% ID/g ± SEM) for *in vivo* PET imaging and *ex vivo* gamma counting analysis

Treatment	Time point	PET/CT*	Gamma counting**
0.03 mg/kg PF-07062119	Day 4	4.10 ± 0.16	6.17 ± 0.82
0.03 mg/kg PF-07062119	Day 9	4.48 ± 0.30	8.67 ± 0.54
0.1 mg/kg PF-07062119	Day 4	3.95 ± 0.27	5.22 ± 0.40
0.1 mg/kg PF-07062119	Day 9	6.76 ± 0.41	11.3 ± 1.43
1 mg/kg PF-07062119	Day 4	4.24 ± 0.24	4.70 ± 0.91
1 mg/kg PF-07062119	Day 9	6.18 ± 0.37	13.0 ± 1.10
1 mg/kg PF-07079699 (control)	Day 4	4.10 ± 0.16	5.17 ± 0.43
1 mg/kg PF-07079699 (control)	Day 9	4.31 ± 0.39	7.98 ± 1.22

### *Ex Vivo* Gamma Counting Confirms Imaging

Measurement of radioactivity by gamma counting of excised tumors following PET imaging confirmed the *in vivo* tumor differences between day 4 and day 9 for the two highest dose levels of PF-07062119 (0.1 and 1 mg/kg) and for the control BsAb-treated mice (Fig. 1B). This also revealed the magnitude of difference elicited at day 9 by the highest dose of PF-07062119 compared to the control BsAb PF-07069699 (13.0 ± 1.10 %ID/g ± SEM vs 8.0 ± 1.22 %ID/g ± SEM; *p* = 0.026). In addition, significantly higher radioactivity uptake was observed for the 1 mg/kg vs. 0.1 mg/kg doses of PF-07062119 when comparing the day 9 and day 4 groups (*p* = 0.002 and *p* < 0.001, respectively). Finally, comparison of radioactivity uptake between groups at day 9 revealed significant differences between the lowest and highest dose levels of PF-07062119 (0.1 and 1 mg/kg), *p*= 0.013. Whole blood counts remained consistently low with a measurement of 1.505 ± 0.109 %ID/g (SEM) on day 9 for the highest dose of PF-07062119. Likewise, the muscle uptake averaged 0.468 ± 0.154 %ID/g (SEM), leading to clearly distinct tumor to blood ratios (>8) and tumor to muscle ratios (>27) observed for the highest dose at day 9, as shown in Table 2.

**Table 2.** Selected tissue distribution and tumor to background ratios at Day 9, expressed as percent injected dose per gram (% ID/g ± SEM) from *ex vivo* gamma counting analysis

Treatment	Blood	Muscle	Tumor	Tumor/blood ratio	Tumor/muscle ratio
0.03 mg/kg PF-07062119	2.03 ± 0.16	0.61 ± 0.10	8.67 ± 0.54	4.3	14.1
0.1 mg/kg PF-07062119	1.78 ± 0.15	0.54 ± 0.16	11.3 ± 1.42	6.3	20.9
1 mg/kg PF-07062119	1.51 ± 0.11	0.47 ± 0.15	13.0 ± 1.10	8.6	27.8
1 mg/kg PF-07079699 (control)	2.12 ± 0.22	0.49 ± 0.18	7.98 ± 1.22	3.8	16.5

In general, the *in vivo* PET imaging and *ex vivo* gamma counting correlated well. However, the *in vivo* PET imaging probably underestimated the presence of the CD8 T cells, most likely due to partial volume effects of high radioactivity confined to a relatively small tumor area and often seen in rodent models [19, 20]. These imaging experiments highlighted the effects of repeat dosing of PF-07062119, which led to an increase in signal due to increased CD8+ T cell recruitment to the tumor. This effect of repeat dosing was confirmed by an additional experiment in which mice were imaged at day 6 (supplementary data). These results showed no statistical difference compared to day 4, even at the highest PF-07062119 dose of 1 mg/kg. In contrast, a significant increase was apparent at day 9 when compared to either day 4 or 6 following a second dose of PF-07062119 administered on day 7 (Supplemental section Figure S3).

In evaluating the dose and timing parameters, selection of an intermediate dose of 0.06 mg/kg was also tested to further explore the dose titration relative to PD over the time course of the study. The results (included in the supplementary) demonstrated tracer uptake at the 0.06 mg/kg dose was similar to the 0.03 mg/kg dose. In this experiment, the highest dose level (1 mg/kg) was repeated to serve as an inter-study control, which also demonstrated the highly reproducible results with the ^89^Zr-Df-IAB22M2C tracer in this model. More details of these additional experiments and results, including *ex vivo* gamma counting, and flow cytometry results can be found in the supplementary section of the manuscript.

### Immunohistochemical Analysis of the CD8+ T Cells Demonstrates Increased T Cell Density with Increasing Dosing and ^89^Zr-Df-IAB22M2C Mb Tumor Uptake

**Fig. 3.**
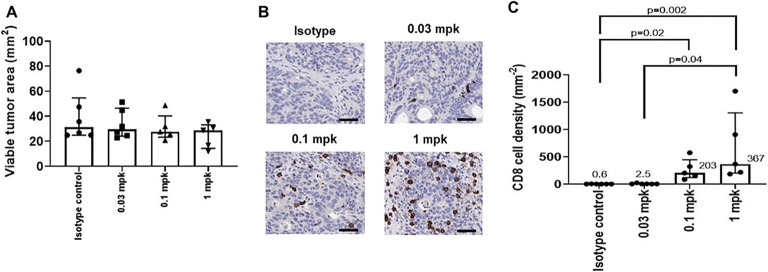
Immunohistochemistry results demonstrating PF-07062119 treatment increases CD8 cell density in tumors at day 9 (cell density mm^2^ mean ± SEM). (**a**) Viable tumor areas demonstrating no significant difference in the viable tumor area amongst groups; therefore, cell density estimates could be compared between groups. (**b**) Immunohistochemistry images of representative slices of tumor at day 9 showing increased CD8+ cells upon dosing. IHC scale bar represents 50 μm. (**c**) The density of CD8+ cells by day 9 increases with increasing treatment dose of PF-07062119 from 0.03, 0.1, and 1.0 mg/kg, with significance being reached for the 0.1 and 1 mpk treated groups relative to vehicle control.

**Fig. 4.**
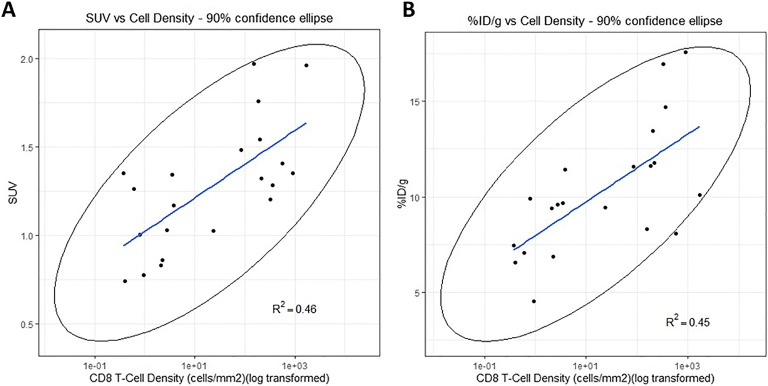
Immunohistochemistry measuring tumor CD8 cell density (log transformed) correlated to (**a**) PET imaging of tumor (SUV) and (**b**) *ex vivo* gamma counting tumor measurements (%ID/g) of the PET tracer for all doses of PF-07062119 treatment at day 9.

Following completion of the imaging studies, tumor tissue was collected for immunohistochemical analysis of the CD8+ T cells. Digital image analysis showed that there was no significant difference in the viable tumor area between the different groups of mice, confirming that cell density estimates could be compared between groups to evaluate effect of treatment (Fig. 3A). The density of CD8+ cells increased with increasing dose levels of PF-07062119 treatment from 0.03, 0.1, and 1.0 mg/kg, significance being reached for the 0.1 and 1 mg/kg treated groups relative to vehicle control (Fig. 3B and C). Moreover, correlations with R^2^ = 0.46 and 0.45, respectively, were observed between radioactivity uptake (SUV or %ID/g) vs. CD8+ IHC staining (cell density/mm^2^) at day 9 in tumor tissues, as illustrated in Fig. 4. The relationship between CD8+ T cell accumulation within the tumor was also assessed by flow cytometry, which revealed a correlation of R^2^ = 0.48 (Supplemental section Figure S4) and confirmed the dose response effect elicited by BsAb therapy. Use of IHC and flow cytometry in this study could provide the key translational links to correlate PET tracer uptake and BsAb treatment with T cell recruitment to the tumor. Although further optimization is required, taken together, these results provide the preliminary basis for qualification of this imaging technique as a pharmacodynamic biomarker.

## Discussion

Immune imaging, or the ability to image the immune response, continues to be a topic of major interest across a variety of disciplines. Here, we report the first reported data evaluating the robustness and sensitivity of a CD8-specific tracer to monitor the pharmacodynamic response of a T cell–bispecific antibody over time and a range of dose levels. As novel immunotherapies continue to expand in clinical oncology, tools that can provide guidance to oncologists regarding tumor response or resistance will be needed to optimize their therapeutic potential. In addition, these tools will guide development of novel IOTs and the expanding use of combination therapies [1]. While ^18^F-FDG PET is a workhorse in cancer management as a glycolytic metabolism biomarker, and evaluation of its utility in IOTs is being explored in systemic immune response and pseudo progression [21], the lack of specific immune–related information remains a challenge and potential limitation. In contrast, by their very nature, anti-CD8 antibodies, of which the 89Zr-Df-IAB22M2C PET tracer is a closely related construct, demonstrate excellent specificity for CD8+ T cells, as reported [16, 22, 23]. Additional information on the tumor micro-environment and specific components of the immune system for monitoring of tumor response to treatment is critical to understanding the course of patient treatment that should be undertaken [24]. Furthermore, biomarkers that can play a role in elucidating an “early response” or “resistance” of tumors would be of great benefit, to patient and physicians alike [25]. In addition to application in clinical treatment, PET immune imaging would be a valuable tool for novel drug development [26]. It could be key to making early strategic decisions, streamlining clinical trials, contributing to overall prioritization of pipelines and their associated combination therapies in the development process.

The potential of redirected T cell therapies has been demonstrated by the approval of blinatumomab in hematological malignancies, and more recently by reports of early clinical activity with CD3 BsAb targeting solid tumors, such as colorectal and prostate cancers [27, 28]. CD3-bispecific antibodies hold potential as potent cancer therapeutics because they recruit and activate a broad repertoire of T cells against tumor cells expressing a tumor-associated cell surface antigen [29]. The use of PET imaging as a supportive, complimentary tool in the non-invasive assessment of CD8+ T cell recruitment and enhancement in solid tumors would be valuable as a pharmacodynamic biomarker [30]. T cell recruitment to tumors, and increases in intratumoral CD8+ T cell numbers, can be either direct or indirect depending on the immunomodulatory strategy employed. This utilization can be expanded more broadly, to include indirect recruitments, or proliferation of tumor-infiltrated CD8+ T cells, such as via a vaccine or virus-based treatment. For this, the CD8+ PET tracer ^89^Zr-Df-IAB22M2C is an attractive tool for monitoring the recruitment, increase, or expansion in the CD8+ T cells, the active cytotoxic T cell that is associated with most tumor death [12, 13].

In order to successfully evaluate the ^89^Zr-Df-IAB22M2C Mb for monitoring CD8+ T cells, the LS1034 adoptive transfer model was selected because it is robust and had produced efficacy with CD3 BsAbs in prior studies [14]. The T-cell engaging CD3-bispecific antibody, PF-07062119 treatment shows dose-dependent efficacy and significant tumor recruitment of engrafted T cells with polarized Granzyme B signal by IHC in this model.

The ^89^Zr-Df-IAB22M2C PET tracer showed substantial uptake in CD8+-rich organs and tissues, namely the spleen and treated tumors, with very little accumulation in background tissues, such as blood or muscle. The CD8 Mb (IAB22M2C, a truncated antibody with a smaller molecular weight (~80 kDa) while maintaining affinity) exhibited very fast clearance from the blood and background tissues at 22 h, a highly desirable property for PET imaging with Zr-89 because it enables next-day imaging [31]. Thus, the pharmacokinetic profile of the anti-CD8 Mb (^89^Zr-Df-IAB22M2C) is favorable for both preclinical and clinical PET imaging, and overcomes limitations with full-length antibodies, which were previously used to image various T cell biomarkers such as CD3, CD4, CD25, or CD45 [32]. The clearance was primarily hepatic in nature, with an additional renal component, as recently described [33]. The PET tracer uptake in tumors demonstrated significant differences between dose levels and days post treatment initiation suggesting ^89^Zr-Df-IAB22M2C PET/CT imaging has the ability to detect and quantify immune reaction following treatments in T cell–engrafted mice. The results were consistent across measurements of *in vivo* PET imaging, *ex vivo* gamma counting, immunohistochemistry, and flow cytometry; together these data underscore the ability of this imaging technique to quantify the dose-dependent pharmacodynamic response to BsAb therapy.

The study was not without its difficulties and confounds. The dynamic changes in T cell infiltration in response to a novel immunomodulatory agent, as well as variability between *in vivo* studies and models, presented challenges in the design. Overall, the very nature of the potentially ever-changing immune target combined with the variability of tumor models has been a challenge for the field [34]. Even with a robust, well-studied model, the sensitivity of the probe needed to be tested, with early timepoints and low BsAb doses that required augmentation once initial results were determined. Based on our initial results, we added a middle dose of the PF-07062119 (0.06 mpk) at an intermediate time point, day 6 (supplementary data – Figure S3). Including this time point in our initial study would have made the study operationally challenging. Lastly, the underestimation of *in vivo* PET imaging compared to *ex vivo* gamma counting was predicted from previous studies as the phenomenon of partial volume effects which occurs when there is high radioactive uptake in small volumes [35]. For this reason, the relationship between IHC and gamma counting is often more informative in preclinical animal models; however, in gamma counting, the full excised tumor is analyzed, while for IHC analysis occurs on a single slice only. As a result, tumor heterogeneity is not well accounted for by the IHC analysis. In addition, the *in vivo* PET quantification is obtained by selecting a region of interest over the tumor region. This tumor region defined on the CT images often does not match perfectly with the excised specimen collected for IHC and gamma counting. Overall, despite these caveats, the CD8+ T cell density by IHC correlation was directionally correct, although relatively low (all data included) with values for both PET imaging (SUV) and gamma counting (%ID/g).

**Fig. 5.**
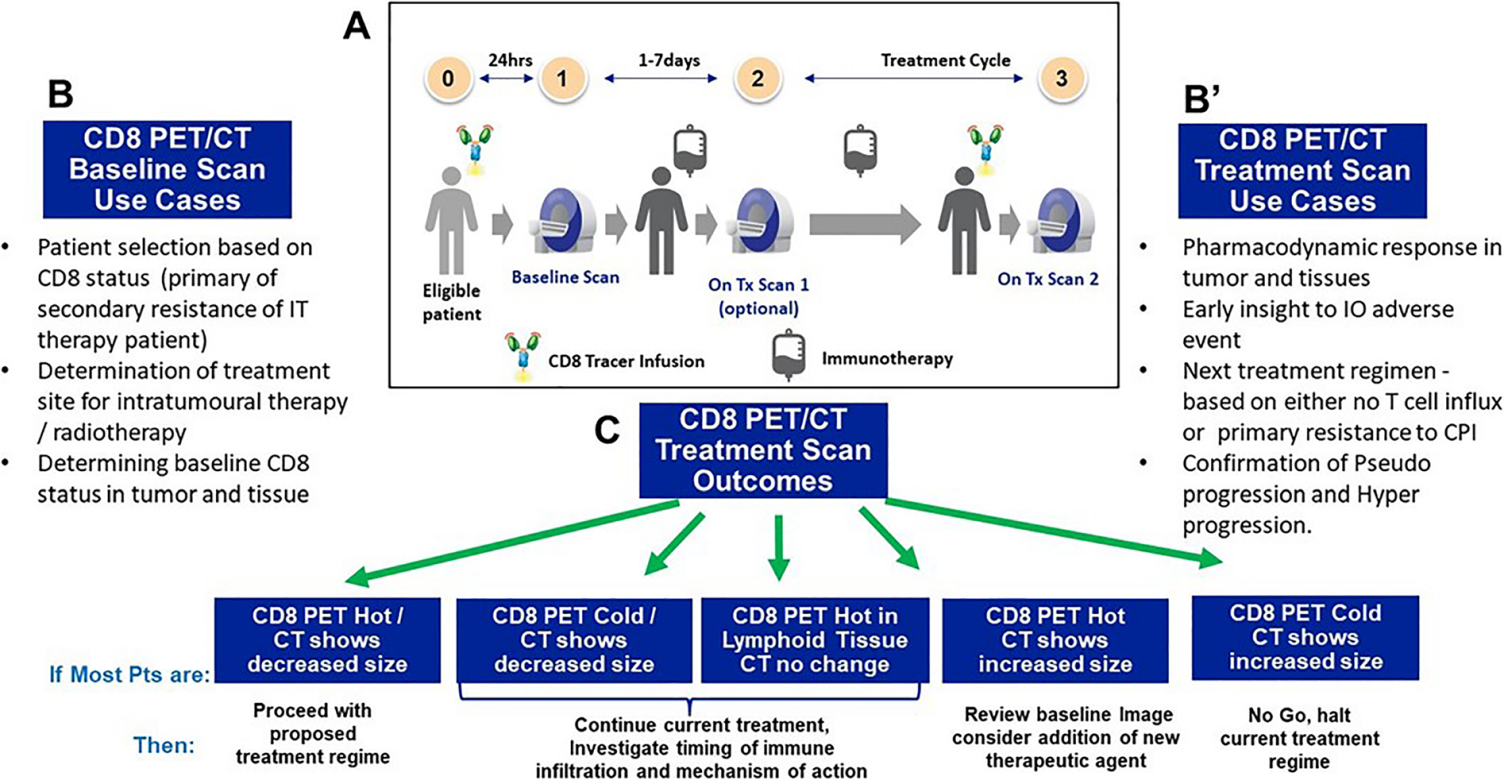
(**a**) The CD8 PET imaging workflow diagram. (**b**) Example use cases for baseline CD8 PET imaging in R&D. (**b’**) Example use cases for post-treatment CD8 PET imaging in R&D. (**c**) The potential clinical decision tree for patients.

In recent literature, there have been a number of reports of promising molecular imaging agents targeting the immune system, including those for activated T cells [36–40], Granzyme B [41, 42], and various PD1 probes [43–45], although many are still in development, or not specific to CD8+ T cells. The CD8 Mb PET technology offers a potential method to quantify tumor-infiltrating lymphocytes as proof of mechanism, complimenting tissue biopsy and standard of care imaging if timing can be optimized to account for observation of critical active T cell populations. The PET tracer, if clinically validated, could provide a biomarker of CD8+ T cell infiltration, well in advance of morphologic changes. Presently, these results are in line with the current ongoing clinical findings, with early clinical results demonstrating that the ^89^Zr-Df-IAB22M2C tracer injection is safe and well-tolerated, and suggests successful targeting of CD8+ T cell–rich tissues [17]. As a marker of treatment effects, CD8 Mb PET imaging is a potential supportive tool for clinical IO drug development [46], with potential applications shown in Fig. 5.

## Conclusion

In this study, we demonstrated the initial steps to qualify the ability of the CD8 Mb PET tracer ^89^Zr-Df-IAB22M2C to quantify the recruitment of human CD8+ T cells to tumors following treatment with the GUCY2C-CD3 BsAb PF-07062119 with high sensitivity and robustness. The PET tracer was able to differentiate over a range of doses and time course and showed a correlation to IHC measurement of CD8+ T cells. While being promising first steps, the PET imaging technique will need further optimization and validation moving forward. In conclusion, we have demonstrated the ability of ^89^Zr-Df-IAB22M2C PET as a preclinical mechanistic biomarker for immune activation measurement to assess the IOT. We believe this radiotracer holds potential as a clinical imaging biomarker for the assessment of CD8+ T cell recruitment. Currently, the ^89^Zr-Df-IAB22M2C PET tracer is being evaluated in phase II clinical testing (NCT03107663) and is a promising tool for monitoring CD8+ T cell biodistributions in patients.

## Supplementary information

ESM 1 (DOCX 2976 kb)

## Abbreviations

%ID/g: Percent injected dose per gram
BsAb: Bispecific antibody
CRC: Colorectal cancer
CT: Computed tomography
CTL: Cytotoxic T lymphocyte
Df: Desferrioxamine
FDG: Fluorodeoxyglucose
FFPE: Formalin fixed paraffin embedded
GUCY2C: Guanylyl cyclase C
HPLC: High-pressure liquid chromatography
IHC: Immunohistochemistry
IO: Immune oncology
IOT: Immune oncology therapies
i.p.: Intraperitoneal
i.v.: Intravenous
mAb: Monoclonal antibody
Mb: Minibody
MIP: Maximum intensity projection
mpk: Milligram per kilogram
PBMC: Peripheral blood mononuclear cell
PD: Pharmacodynamic
PD1/PD-L1: Programmed cell death protein 1/programmed cell death ligand 1
PET: Positron emission tomography
RECIST: Response Evaluation Criteria in Solid Tumors
SD: Standard deviation
SEM: Standard error of the mean
SUV: Standard uptake value
TME: Tumor microenvironment
Tx: Treatment
