# Supplementary material for: Preclinical Evaluation of 89Zr-Df-IAB22M2C PET as an Imaging Biomarker for the Development of the GUCY2C-CD3 Bispecific PF-07062119 as a T Cell Engaging Therapy
Source: Mol Imaging Biol. 2021 Jun 18;23(6):941–51. doi: 10.1007/s11307-021-01621-0 (PMC8578158; doi:10.1007/s11307-021-01621-0)
Supplement: Supplementary file 1 — (DOCX 2976 kb) [file 11307_2021_1621_MOESM1_ESM.docx]

**Electronic Supplementary Material**

**Preclinical Evaluation of ^89^Zr-Df-IAB22M2C PET as an Imaging Biomarker for the Development of the GUCY2C-CD3 Bispecific PF-07062119 as a T Cell Engaging Therapy.**

**“Journal: Molecular Imaging and Biology”**

Kevin P. Maresca^1^, Jianqing Chen^1^, Divya Mathur^1^, Anand Giddabasappa^1^, Adam Root^1^, Jatin Narula^1^, Lindsay King^1^, David Schaer^1^, Jonathan Golas^1^, Keith Kobylarz^1^, Edward Rosfjord^1^, Edmund Keliher^1^, Laigao Chen^1^ , Sripad Ram^1^, Eve H. Pickering^1^, James S. Hardwick^1^, Paul A. Rejto^1^, Amira Hussein^2^, Ohad Ilovich^2^, Kevin Staton^3^, Ian Wilson^4^, Timothy J. McCarthy^1^.

^1^ Worldwide Research, Development & Medicine, Pfizer Inc, USA; ^2^ Invicro, a Konica Minolta company; ^3^ Memorial Sloan Kettering Cancer Center, New York, NY, USA; ^4^ ImaginAb Inc., Inglewood CA, USA.

**Corresponding author:**

Kevin P. Maresca, PhD

E-mail: kevin.maresca@pfizer.com

Phone: 1 978-821-9226

Mail address: 610 Main Street, Cambridge, MA 02139

**Supplementary Materials and Methods**

**Supplementary methods.**

The tumor tissue taken for cell extraction were finely minced with scissors and incubated in a digestion buffer (composed of: RPMI-1640, FBS, Collagenase D, Hyaluronidase and DNase 1). The vials were vortexed and allowed to digest in a 37°C bath on a sample rocker for 40 minutes. After cell dissociation, a pre-wet 70 μm filter was used to collect the cells and rinsed. The cells were rinsed and prepared for staining. The extracted cells were stained for mCD45, hCD45, hCD3, hCD4, and hCD8. The cells were incubated in an antibody cocktail for 30 minutes over ice. The cells were then rinsed and incubated in Zombie NIR (BioLegend, 423106) solution for 10 minutes over ice for viability staining. After washing, the cells were fixed, storage occurred at 4°C for 30 days to allow for radioactive decay.

The flow cytometry panel included the following antibody conjugates to distinguish human from mouse lymphocytes and to identify any changes in human lymphocytes due to treatment – anti-human anti-CD45-FITC (clone HI30, BD Biosciences, 555482), anti-human anti-CD3-PE-Cy7 (clone SK7, BioLegend, 344816), anti-human anti-CD4-BV421 (clone RPA-T4, BioLegend, 300532), anti-human anti-CD8-APC (clone RPA-T8, BD Biosciences, 555369), and anti-mouse anti-CD45-BV711 (clone 30-F11, BioLegend, 103147).  These fluorophore conjugated antibodies were used in an antibody mastermix to label input T cells, as well as the harvested tumors. Tumors were dissociated into single cell suspensions using a tumor dissociation kit (Miltenyi 130-096-730) according to the manufacturer’s guidelines.

Cell suspensions were washed and then stained with the antibody mastermix with 1-3 million cells in 100 µL of staining buffer (PBS (Ca/Mg free), 0.5% BSA, 5% normal mouse serum).  Cells were incubated at room temperature for 30 minutes before being washed and resuspended in PBS (Ca/Mg free).  Viability staining was performed with a 1:1000 dilution of Zombie NIR dye for ten minutes in 0.1 mL of PBS (Ca/Mg free).  After washing in staining buffer, cells were fixed in 2% paraformaldehyde (Electron Microscopy Sciences) overnight at 4°C.  Cells were pelleted and resuspended in freezing media containing 90% FBS and 10% DMSO and stored at -80ºC until acquisition. Prior to acquisition, samples were thawed at 37 °C and washed in Staining Buffer.  The concentration of the cell suspension was adjusted so samples are acquired at a rate of 250 to 500 events per second. Flow cytometric data were acquired on a BD Biosciences Fortessa X-20 flow cytometer using BD Bioscience’s FACSDiva software version 8.0.1.   FCS (Flow Cytometry Standard) raw data files were uploaded to FCS Express version 7.0 (DeNovo Software) for manual gating and phenotyping analysis. Lymphocyte populations were analyzed using GraphPad Prism version 8.4.2 software.

**Supplementary Fig. S1.** Representative immunohistochemistry time course of CD3 and Granzyme B for the human CRC LS1034 tumors treated with PF-07062119.

**Supplementary Fig. S2.** Experimental scheme displaying the treatment schedule and imaging timepoints for the BsAb CD8 PET study.

**Supplementary Fig. S3.** Gamma counting of tumors of LS1034 mice (mean %ID/g ± SEM) at 22h post ^89^Zr-Df-IAB22M2C injection on Day 6 and 9: 0.06, 0.1 and 1 mg/kg GUCY2C-CD3 BsAb (PF-07062119) and Isotype (PF-07069699) treated.

**Supplementary Fig. S4.** Flow cytometry CD8 cell density measurement correlation to *ex vivo* tumor tissue gamma counting measurements (%ID/g) of the PET tracer at all time points and all doses of GUCY2C-CD3 BsAb (PF-07062119) Treatment, represented as group averages.


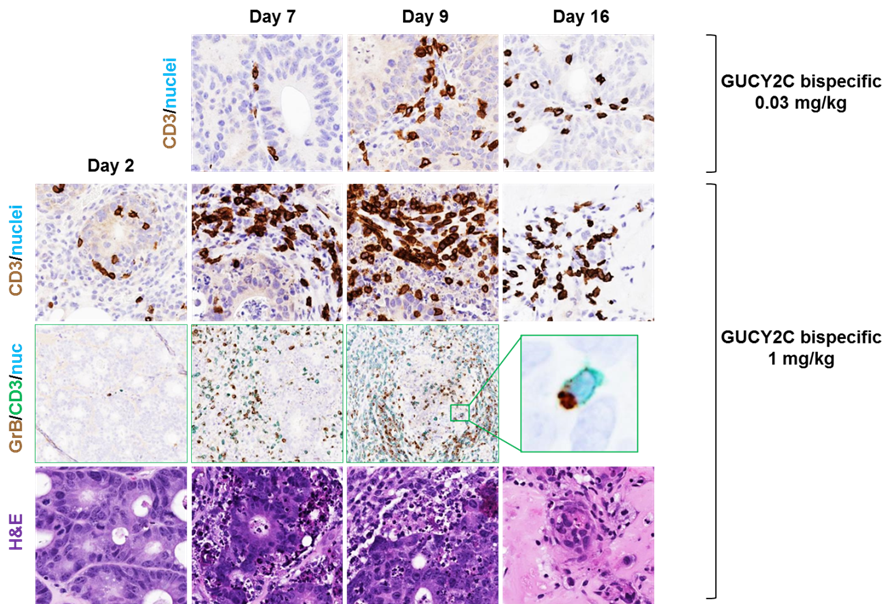


**Day 2**

**Day 7**

**Day 9**

**Day 16**

PF-07062119 – 1 mg/kg

**CD3 and Granzyme B IHC**

**Figure S1.** Representative immunohistochemistry time course of CD3 and Granzyme B for the human CRC LS1034 tumors implanted into NOD scid gamma (NSG) female mice treated with PF-07062119.

**
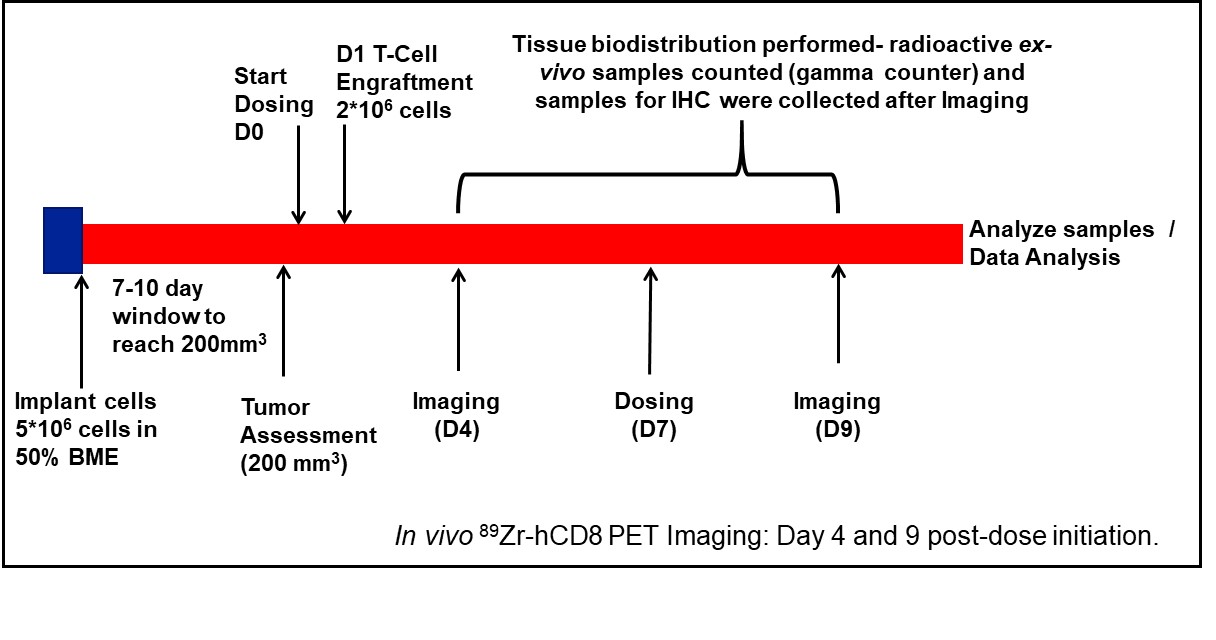
**

**Figure S2.** Experimental scheme displaying the treatment schedule and imaging timepoints for the BsAb CD8 PET study.

**Figure S3.** Gamma counting of tumors of LS1034 mice (mean %ID/g ± SEM) at 22h post ^89^Zr-Df-IAB22M2C injection on Day 6 and 9: 0.06, 0.1 and 1 mg/kg GUCY2C-CD3 BsAb (PF-07062119) and Isotype (PF-07069699) treated.

**Figure S4.** Flow cytometry CD8 cell density measurement correlation to *ex vivo* tumor tissue gamma counting measurements (%ID/g) of the PET tracer at all time points and all doses of GUCY2C-CD3 BsAb (PF-07062119) Treatment, represented as group averages.
